# Lamin A/C-dependent interaction with 53BP1 promotes cellular responses to DNA damage

**DOI:** 10.1111/acel.12258

**Published:** 2015-01-23

**Authors:** Ian Gibbs-Seymour, Ewa Markiewicz, Simon Bekker-Jensen, Niels Mailand, Christopher J Hutchison

**Affiliations:** 1 School of Biological and Biomedical Sciences, Durham UniversityMountjoy Science Park, Durham, DH1 3LE, UK; 2 Ubiquitin Signaling Group, Department of Disease Biology, Novo Nordisk Foundation Center for Protein Research, University of CopenhagenCopenhagen, DK-2200, Denmark

**Keywords:** 53BP1, DNA damage response, lamins, laminopathy

## Abstract

Lamins A/C have been implicated in DNA damage response pathways. We show that the DNA repair protein 53BP1 is a lamin A/C binding protein. In undamaged human dermal fibroblasts (HDF), 53BP1 is a nucleoskeleton protein. 53BP1 binds to lamins A/C via its Tudor domain, and this is abrogated by DNA damage. Lamins A/C regulate 53BP1 levels and consequently lamin A/C-null HDF display a 53BP1 null-like phenotype. Our data favour a model in which lamins A/C maintain a nucleoplasmic pool of 53BP1 in order to facilitate its rapid recruitment to sites of DNA damage and could explain why an absence of lamin A/C accelerates aging.

## Introduction

The integrity of genomic DNA is constantly under threat from both endogenous and exogenous sources of damage, with the double-strand break (DSB) being one of the most cytotoxic lesions (Bekker-Jensen & Mailand, 2010; Ciccia & Elledge, 2010). The DNA damage response (DDR) is a complex signal transduction pathway that is essential for preserving genomic DNA and acts as part of an antitumourigenesis barrier (Jackson & Bartek, 2009) as well as being a critical driver of aging (Passos *et al*., 2009).

53BP1 is a DDR protein that plays an important function in preserving genomic integrity. 53BP1 is recruited to DSBs via a bivalent recognition of dimethylated histone H4K20 (K4K20me2) and histone H2A ubiquitylated on lysine 15 (Fradet-Turcotte *et al*., 2013). Functionally, 53BP1 has been shown to govern the mechanisms that are required to repair cellular DSBs. Most DSBs are repaired by either homologous recombination (HR) or nonhomologous end joining (NHEJ), and 53BP1 is central to the cellular decision to engage the HR or NHEJ pathways (Bothmer *et al*., 2010; Bunting *et al*., 2010; Chapman *et al*., 2012). 53BP1 is an inhibitor of DNA 5′ end resection, an early step of HR, thereby promoting NHEJ as the repair choice (Bothmer *et al*., 2010; Bunting *et al*., 2010). 53BP1 has also been shown to maintain genomic stability by facilitating DNA repair of DSB ends by promoting chromatin mobility (Dimitrova *et al*., 2008).

Laminopathies are genetic diseases caused by mutations in the gene *LMNA*, which encodes nuclear lamins A/C, or in genes encoding proteins that interact with lamins A/C (Broers *et al*., 2006). The most severe laminopathies are progeroid syndromes, including Hutchinson–Gilford progeria syndrome (HGPS), which often but not exclusively arise as a result of incomplete post-translational processing of either prelamin A or a variant form of lamin A termed progerin (Eriksson *et al*., 2003). Cells from HGPS patients are characterized by the accumulation of abnormally shaped nuclei, accumulation of DNA damage and premature senescence (Goldman *et al*., 2004; Liu *et al*., 2005; Scaffidi & Misteli, 2006). Mechanistically, it is unclear how defects in the nuclear lamina impinge on the DDR; however, *Lmna*^*−/−*^ MEFs are deficient in 53BP1 and are unable to carry out NHEJ (Gonzalez-Suarez *et al*., 2009).

With this in mind, we investigated the association between lamins A/C and 53BP1. We show that lamins A/C are 53BP1-binding proteins that promote 53BP1 nuclear retention via the latter's Tudor domain in a DNA damage and ataxia telangiectasia mutated (ATM)-dependent mechanism. Human cells deficient for lamins A/C are defective in DNA repair and exhibit impaired cellular survival in response to DNA damage, underlining their importance in genome stability.

## Results and discussion

### 53BP1 cofractionates with lamins A/C in undamaged HDF

**Figure 1:**
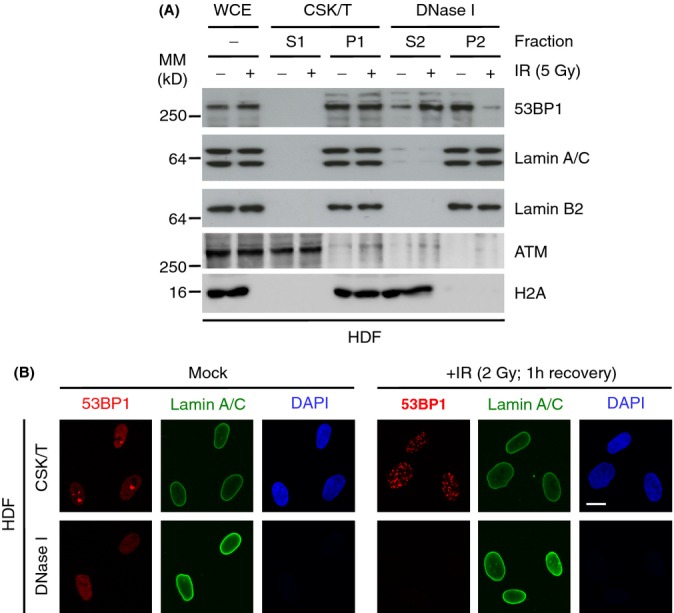
53BP1 cofractionates with lamins A/C in HDF. (A) HDF were treated with IR (5 Gy, 1 h) and fractionated by sequential treatments of detergent (CSK/T) and DNase I. Extracts were then processed for immunoblotting using the indicated antibodies. WCE, whole cell extract. P, pellet; S, supernatant. (B) HDF were treated with IR (2 Gy; 1 h) and extracted *in situ* with CSK/T followed by DNase I digestion before being processed for immunofluorescence using lamin A/C and 53BP1 antibodies. Scale bar, 10 μm.

To understand its relationship to the nuclear lamina, we used a nucleoskeleton extraction procedure on human dermal fibroblasts (HDF) to investigate the biochemical properties of 53BP1. Human dermal fibroblasts were extracted sequentially with Triton X-100, DNase I and (NH_4_)_2_SO_4_ to generate soluble, chromatin and nucleoskeleton fractions. In undamaged HDF the majority of 53BP1 was in the nucleoskeleton and cofractionated with lamins. As expected, histone H2A was soluble after DNase I extraction, whilst a second DDR protein ATM was also soluble. Following ionizing radiation (IR), the behaviour of H2A did not change, whilst a small fraction of ATM became more insoluble. The biochemical properties of the lamins did not change following IR. In contrast, 53BP1 ceased to behave as a nucleoskeleton protein and instead cofractionated with chromatin (Fig.1A). Fluorescence microscopy also showed that in undamaged HDF, 53BP1 had a granular distribution throughout the nucleoplasm and this distribution was retained following nucleoskeleton extraction. In contrast, following IR 53BP1 was confined to DNA damage foci which were completely solubilized following DNase I digestion (Fig.1B). The retention of the nucleoskeleton protein NuMa following DNase I digestion in both undamaged (not shown) and IR-treated HDF (Fig. S1A) confirmed that the nucleoskeleton *per-se* was not disrupted by DNA damage. Thus 53BP1 is protected like a nucleoskeleton protein in undamaged HDF but is unprotected like a chromatin binding protein after IR.

### 53BP1 Tudor domain is required for interaction with lamin A

**Figure 2:**
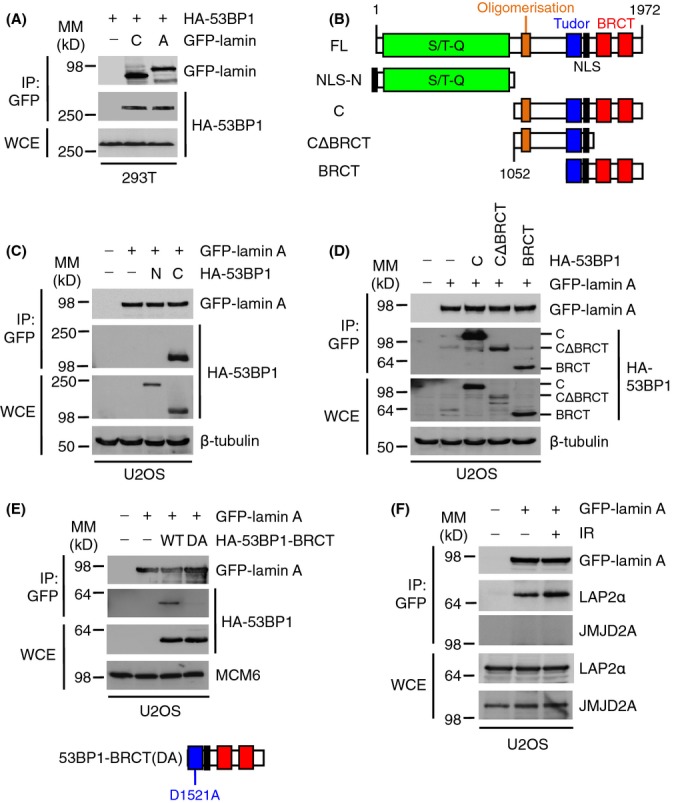
Lamins A/C-53BP1 interaction is dependent on the 53BP1 Tudor domain. (A) HEK293T cells were cotransfected with HA-53BP1 and either GFP-empty vector (−), GFP-lamin A or GFP-lamin C. Cell extracts were then subjected to immunoprecipitation using GFP-Trap beads, and bound complexes were then analysed by immunoblotting using HA and GFP antibodies. WCE, whole cell extract, IP: immunoprecipitates. WCE represents 1% input. (B) HA-53BP1 constructs used in this study. FL, full length. NLS, nuclear localization signal. (C) U2OS/GFP-lamin A cells transfected with HA-53BP1 N or C constructs for 24 h were subjected to GFP immunoprecipitation followed by immunoblotting with HA and GFP antibodies. β-tubulin was a loading control. WCE represents 1% input. (D) U2OS/GFP-lamin A cells were transfected with HA-53BP1 C-terminal constructs for 24 h and processed as in (C). WCE represents 1% input. (E) U2OS/GFP-lamin A cells were transfected with HA-53BP1-BRCT wild-type (WT) or D1521A (DA) mutant for 24 h and processed as in (C). MCM6 was used as a loading control. WCE represents 1% input. (F) U2OS/GFP-lamin A cells were subjected to IR (10 Gy) and allowed to recover for 1 h. Cell extracts were then subjected to immunoprecipitation using GFP-Trap beads, and bound complexes were then analysed by immunoblotting using GFP, LAP2α and JMJD2A antibodies. WCE represents 1% input.

To understand the behaviour of 53BP1, we used co-immunoprecipitation to investigate its interactions with lamins A/C. First, we cotransfected 293T cells with HA-tagged 53BP1 and GFP, GPF-lamin A or GFP-lamin C and immunoprecipitated cell lysates with anti-GFP anitbodies. HA-53BP1 interacted efficiently and equally with GFP-lamin A and GFP-lamin C, but not with GFP-empty vector (Fig.2A). Next, we transfected U2OS/GFP-lamin A cells (Fig. S1B,C) with a series of deletion constructs encoding different fragments of HA-tagged 53BP1 (Fig.2B). A C-terminal fragment of 53BP1 co-immunoprecipitated with GFP-lamin A, whereas an N-terminal fragment did not interact with lamin A at all (Fig.2C). To further define the site of interaction between lamin A and 53BP1, we investigated its interaction with three C-terminal fragments, one encoding the entire C-terminal domain, one lacking the oligomerization domain and one lacking the BRCT domain. All three fragments interacted efficiently with GFP-lamin A (Fig.2D). As all of these fragments contained the Tudor domain, our results implied that lamin A interacts with 53BP1 via its Tudor domain. To confirm this, we created a D1521A mutation within the Tudor domain (that eliminates binding H4K20me2) of the BRCT fragment (Botuyan *et al*., 2006). The mutant inhibited the interaction between the fragment and GFP-lamin A, suggesting that the Tudor domain of 53BP1 is required for lamin A interaction (Fig.2E). As positive and negative controls, we also investigated the interaction between lamin A and the nucleoskeleton protein LAP2α or the DDR protein JMJD2A, the latter also contains a Tudor domain (Chen *et al*., 2011). LAP2α bound efficiently to lamin A in extracts prepared from both undamaged and IR-treated cells. In contrast, JMJD2A did not bind to lamin A under either condition (Fig.2F).

### DNA damage-dependent abrogation of the interaction between lamin A and 53BP1

**Figure 3:**
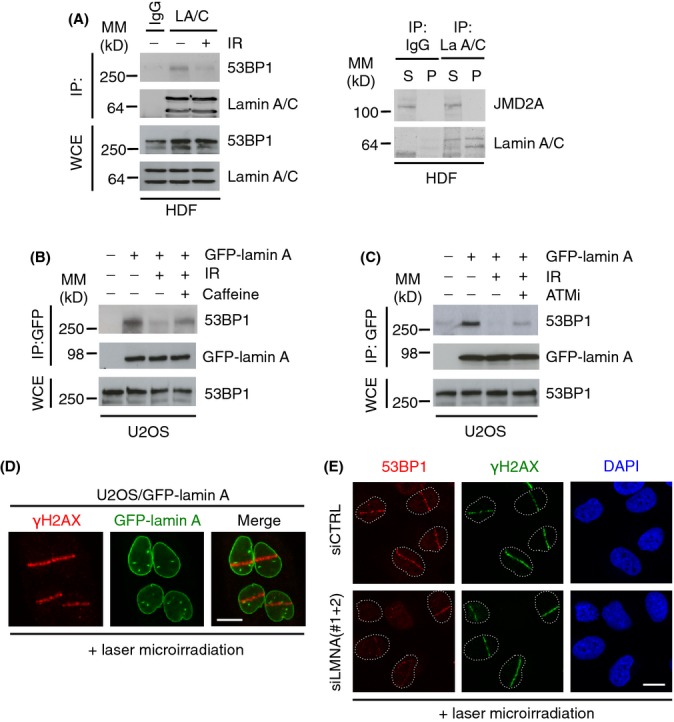
Lamin A/C-53BP1 interaction is regulated in a DNA damage-dependent manner. (A) *Left;* HDF were subjected to IR (10 Gy) and allowed to recover for 1 h. Association between A-type lamins and 53BP1 was assessed by immunoprecipitation of endogenous lamin A/C followed by immunoblotting with 53BP1 and lamin A/C antibodies. WCE, whole cell extract, IP: immunoprecipitates. WCE represents 5% input. *Right;* endogenous lamin A/C was immunoprecipitated and supernatants or pellets were analysed by immunoblotting for interaction with JMJD2A. (B) U2OS/GFP-lamin A cells were pretreated with caffeine (20 mm) for 1 h before exposure to IR (10 Gy, 1 h recovery). Cell extracts were then subjected to immunoprecipitation using GFP-Trap beads, and bound complexes were then analysed by immunoblotting using 53BP1 and GFP antibodies. WCE represents 1% input. (C) As in (C) except cells were pretreated with 10 μm ATMi for 1 h before IR. WCE represents 1% input. (D) U2OS/GFP-lamin A cells were subjected to laser micro-irradiation, fixed 1 h later and immunostained with γ-H2AX antibody. Scale bar, 10 μm. (E) U2OS cells were transfected with siCTRL or siLMNA and subjected to laser micro-irradiation, fixed 15 min later and then processed for immunofluorescence with γ-H2AX and 53BP1 antibodies. Scale bar, 10 μm.

Next, we investigated whether the endogenous lamin A/C-53BP1 interaction in HDF was IR-dependent. Whilst 53BP1 co-immunoprecipitated with lamins A/C from extracts of undamaged cells, this interaction was substantially reduced following IR (Fig.3A). Furthermore, as for GFP-lamin A, endogenous lamin A/C also did not interact with the Tudor domain-containing protein JMJD2A (Fig.3A).

53BP1 is a target for ATM-dependent phosphorylation, which is important for its *in vivo* functions, one of which is to recruit RIF1 to sites of DSBs to inhibit end resection (Chapman *et al*., 2013; Di Virgilio *et al*., 2013; Escribano-Diaz *et al*., 2013; Zimmermann *et al*., 2013). To investigate whether loss of 53BP1-lamin A/C interaction following irradiation is due to ATM phosphorylation, we pre-incubated U2OS/GFP-lamin A cells with either caffeine or the ATM inhibitor (ATMi) KU55933. In the presence of caffeine or ATMi, the post-IR interaction of lamins A and 53BP1 was partially restored (Fig.3B,C). These data show that the 53BP1-lamin A/C interaction is disrupted by DNA damage-dependent ATM phosphorylation. We then investigated whether the DNA damage-dependent 53BP1-lamin A/C association was also mediated through the Tudor domain. Ionizing radiation had no effect on the interaction between GFP-lamin A and HA-53BP1 BRCT (Fig. S2A) indicating that, whilst the Tudor domain is necessary for 53BP1-lamin interactions, sequences N-terminal to the Tudor domain of 53BP1 are needed to infer DNA damage sensitivity to this interaction.

Next, we investigated whether loss of A-type lamins might influence 53BP1-dependent DNA damage pathways. Initially, we used laser micro-irradiation to induce DNA damage in U2OS cells (Bekker-Jensen *et al*., 2006). Importantly, lamin A is not recruited to sites of DNA damage induced by laser micro-irradiation or IR treatment showing that it is not directly involved in the DDR (Figs3D and S2B). In U2OS cells treated with control siRNA, recruitment of 53BP1 to sites of laser irradiation was very efficient. In contrast, in cells treated with siRNA against lamins A/C, recruitment of 53BP1 to sites of DNA damage exhibited weak accumulation in most cells (Fig.3E). Importantly, following siRNA knockdown of lamin A/C in these experiments was very efficient, whereas 53BP1 levels persisted (Fig. S2C), suggesting that failure to recruit 53BP1 to sites of DNA damage was a direct consequence of lamin A/C deficiency.

### Loss of lamin A/C inhibits DNA repair and causes genomic instability

**Figure 4:**
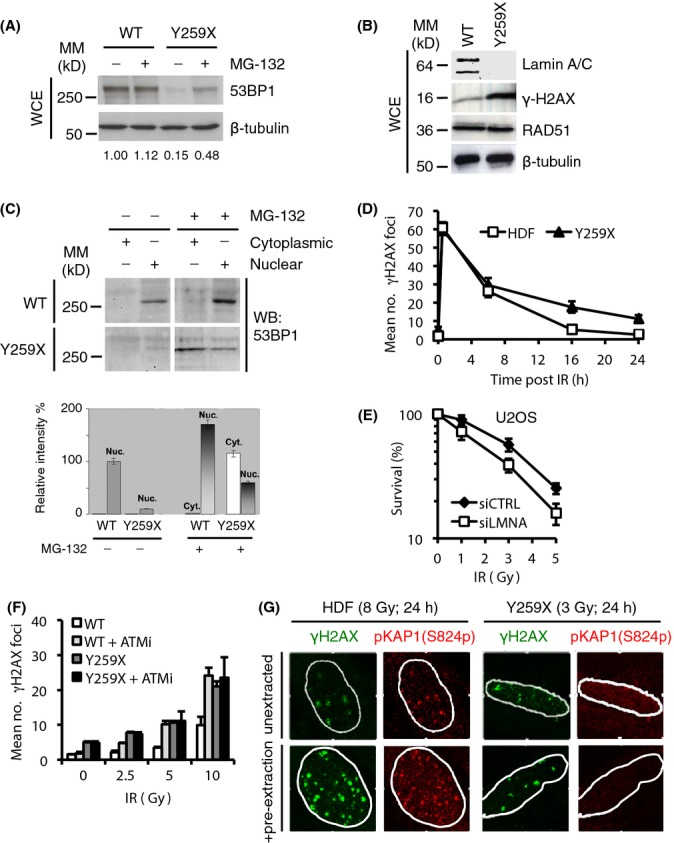
A-type lamin depletion inhibits DNA repair and reduces cellular fitness to DNA damage. (A) Wild-type (WT) or Y259X HDF were treated with the proteasome inhibitor MG-132 (30 μm). Cell extracts were prepared and levels of 53BP1 were assessed by immunoblotting. β-tubulin was a loading control. WCE, whole cell extract. The relative levels of 53BP1 were expressed relative to the levels of untreated WT HDF (1.00). In untreated Y259X cells, 53BP1 levels fell to 15% and were restored to ∽50% following treatment with MG-132. (B) Cell extracts were prepared from WT and Y259X HDF and assessed by immunoblotting with the indicated antibodies. Shown is a representative set of blots from two independent experiments. (C) WT and Y259X HDF were biochemically extracted into cytoplasmic and nuclear fractions before immunoblotting for levels of 53BP1. (D) HDF and Y259X cells were treated with IR (3 Gy), fixed at the times indicated and stained with γ-H2AX antibody. The number of γ-H2AX foci at each time point was quantified and data represents the mean ± SD from three independent experiments. (E) U2OS cells were transfected with siCTRL or siLMNA siRNA and plated onto 60-mm dishes. The cells were then exposed to varying doses of IR and fixed 12 days later in a solution containing crystal violet. The surviving fraction at each IR dose was calculated based on the plating efficiency of the untreated plates. Data represent the mean ± SD from three independent experiments. (F) HDF or Y259X cells were treated with DMSO or KU55933 ATM inhibitor (10 μm) before exposure to the indicated doses of IR then fixed, processed and stained with γ-H2AX antibody. Residual γ-H2AX foci were enumerated and the data represent the mean ± SD from three independent experiments. (G) HDF or Y259X cells were exposed to IR, briefly extracted with CSK buffer and processed for immunofluorescence with γ-H2AX and pKAP-1 antibodies. Scale bar, 10 μm.

It has previously been shown that in Lmna^−/−^ MEFs, 53BP1 protein is unstable and that loss of 53BP1 protein leads directly to an inability to perform NHEJ (Redwood *et al*., 2011). We therefore investigated whether 53BP1 protein expression is unstable in Y259X lamins A/C-null human fibroblasts (Pekovic *et al*., 2011). We found that in the absence of lamins A/C, 53BP1 protein levels were greatly reduced, whereas other DNA damage proteins such as RAD51 were unaffected, and the DDR marker γ-H2AX was significantly increased (Fig.4A,B), reflecting accumulated DNA damage in these cells. To show that the loss of 53BP1 was a consequence of loss of lamin A/C expression, we found that lamins A/C silencing in HDF led to greatly reduced levels (50%) of 53BP1 (Fig. S3A). Previously, we showed that lamins A/C silencing in human cells leads to cell cycle arrest (Pekovic *et al*., 2007). To ensure that loss of 53BP1 accompanying lamin A/C silencing was not an indirect consequence of cell cycle arrest, we induced either quiescence or senescence in HDF and measured 53BP1 levels. We found that 53BP1 expression was insensitive to either temporary or permanent cell cycle arrest (Fig. S3B). Finally, it has been reported that in *Lmna*^−/−^ MEFs, 53BP1 is susceptible to degradation via the proteasome (Gonzalez-Suarez *et al*., 2009). To investigate the cause of 53BP1 loss, we treated lamin A/C silenced HDF or Y259X fibroblasts with the proteasome inhibitor MG-132. We found that in the presence of MG-132 and the absence of lamins A/C, levels of 53BP1 expression were partially restored (Figs4A and S3A). Degradation of 53BP1 in the absence of lamins A/C appeared to occur because it was not retained in the nucleus as treatment of Y259X fibroblasts with MG-132 led to a distribution of 53BP1 between the nucleus and cytoplasm (Fig.4C).

To understand the consequence of loss of 53BP1 in Y259X fibroblasts, we induced DSBs in HDF by IR and assessed rates of DNA repair using γ-H2AX as a marker (Fig. S3C). We found that the fast phase of repair (usually indicative of euchromatic repair – Goodarzi & Jeggo, 2012) was identical in both wild-type and Y259X HDF. In contrast, the slow phase of repair was significantly impaired in Y259X fibroblasts leading to increased DNA damage foci (Fig.4D). In U2OS cells, this accumulation of DNA damage foci (not shown) was correlated with reduced levels of cell survival in response to increasing doses of IR (Fig.4E).

To confirm that the impaired DDR in Y259X fibroblasts results from a 53BP1 deficiency, we initially attempted to over-express 53BP1 in these cells using a retroviral vector. However, Y259X cells are very fragile (Pekovic *et al*., 2011) and transfection even with an empty vector led to widespread cell death. It has been shown that 53BP1 facilitates ATM-mediated DSB repair via activation of the heterochromatin building factor KRAB-associated protein 1 (KAP-1) (Noon *et al*., 2010). In this study, a signature of 53BP1 deficiency included insensitivity to ATMi and an absence of phosphorylated KAP-1 (pKAP-1) at γ-H2AX repair foci. To confirm that the DNA damage repair defect observed in Y259X fibroblasts was due to loss of 53BP1, we investigated the effects of ATMi on the generation of DSBs. We found that whilst levels of DSBs in HDF were sensitive to and increased dramatically in the presence of ATMi, DSB accumulation in Y259X fibroblasts was insensitive to ATMi (Fig.4F), similar to 53BP1 deficient cells (Noon *et al*., 2010). Similarly, whilst pKAP-1 foci formed readily and were detergent insoluble in HDF and some (∽15%) colocalized with γ-H2AX foci, pKAP-1 foci were almost absent from Y259X fibroblasts and pKAP-1 was completely soluble following detergent extraction (Fig.4G) even though total KAP-1 levels were unaffected (Fig. S3D). These data suggest that accumulation of DSBs and cell death in the absence of A-type lamins, like in *Lmna*^−/−^ MEFs, is at least in part, caused by loss of 53BP1.

We have shown that 53BP1 interacts with lamins A/C in a DNA damage- and ATM-dependent manner, and this interaction is dependent upon the integrity of 53BP1 Tudor domain. Human dermal fibroblasts cells null for lamin A/C exhibit reduced 53BP1 protein levels due to proteasomal degradation and as a consequence have a DNA damage phenotype that overlaps with 53BP1 null cells (Noon *et al*., 2010) including increased sensitivity to IR, inhibition of the slow phase of DSB repair, loss of sensitivity to ATMi and an absence of pKAP-1 from sites of DSBs. In contrast, following siRNA knockdown of lamin A/C is U2OS cells, 53BP1 persists but is not recruited to sites of DNA damage. Our results build on recent work that has shown a function for A-type lamins in the DDR. In *Lmna*^−*/*−^ MEFs, 53BP1 protein levels are reduced by a combination proteasomal degradation and upregulated expression of the cysteine protease cathepsin L (CTSL), which in turn directly impairs classical-NHEJ (Gonzalez-Suarez *et al*., 2011; Redwood *et al*., 2011). The finding is entirely consistent with our finding that MG-132 only partially restores 53BP1 levels in human Y259X cells and our data that the slow phase of DNA repair is compromised in Y259X cells. Our finding that siRNA knockdown of lamin A/C leads to impaired recruitment of 53BP1 to sites of DNA damage in U2OS cells when significant 53BP1 levels persisted (Figs3F and S2C) implies that lamin A/C might influence DNA damage repair through two distinct mechanisms. In HDF, lamin A/C directly affects 53BP1 stability and therefore function. In U2OS cells, it affects recruitment. It is known that lamin A/C affects chromatin architecture, which in turn is necessary for ATM activation (Kim *et al*., 2009). Thus, we speculate that lamin A/C might also affect 53BP1 function via an ATM response that relies upon chromatin modelling.

The site of lamin A/C interaction with 53BP1 maps to fragments containing the Tudor domain and is abrogated by a Tudor domain point mutant D1521A, which also abolishes 53BP1 interaction with H4K20me2 at sites of DNA damage (Botuyan *et al*., 2006). Whether the 53BP1-lamin A/C interaction is direct or part of a larger protein complex is not known. However, a recent proteomic study that measures nearest neighbour interactions did identify 53BP1 as a direct binding partner of lamin A (Roux *et al*., 2012). Regardless, our data imply that ATM-mediated phosphorylation events, of 53BP1 and other targets (Matsuoka *et al*., 2007), may disrupt this interaction. In addition, the DNA damage-dependent chromatin remodelling may increase the availability of H4K20me2, promoting dissociation between 53BP1 and lamin A/C (Botuyan *et al*., 2006). These combined findings lead us to hypothesize that lamin A/C-53BP1 interactions are important for either its (53BP1) retention within the nucleus or its activation via ATM or both.

One speculative consequence of our findings is that the lamin A/C-53BP1 interaction is potentially important for entry into a senescent state, particularly in human fibroblasts. Lamin A/C deficiency in HDF causes premature senescence, which is characterized by a defective DDR (Pekovic *et al*., 2011). Thus, it is possible that signalling through 53BP1 that induces cellular senescence could occur via either persistent DNA damage or defective A-type lamin organization, or both (Hutchison, 2012). As not all human cells express A-type lamins (e.g. lymphoblastoid cells), this does raise questions as to how 53BP1 function could be maintained in cells naturally deficient in A-type lamins. We speculate that in these cells either CTSL regulation is not lamin-dependent or that 53BP1 nuclear anchorage or activation is achieved through other mechanisms.

## Materials and methods

### Plasmids and siRNA

Plasmids GFP-lamin C and GFP-lamin A have been described (Pekovic *et al*., 2007). HA-53BP1 constructs used were as described (Iwabuchi *et al*., 2003). The D1521A point mutation was introduced using the QuikChange Site Directed Mutagenesis Kit (Stratagene, Agilent Technologies, Cheshire, UK) and verified by sequencing. Plasmid transfections were performed using GeneJuice (Novagen, Merck Millipore, Watford, UK) according to the manufacturer's guidelines. siRNA transfections were performed using Lipofectamine RNAiMax (Invitrogen, Life Technologies, Paisley, UK) according to the manufacturer's guidelines. Oligonucleotides for siRNA were lamin A/C #1 (5′-CUGGACUUCCAGAAGAACA-3′) and lamin A/C #2 (5′-GCAACUUCAGGAUGAGAUG-3′).

### Cell culture and reagents

Human dermal fibroblasts used have been described previously (Pekovic *et al*., 2011). Human dermal fibroblasts, U2OS and 293T cells were cultured in DMEM containing 10% FBS (Sigma, Gillingham, UK). Human dermal fibroblasts from a patient with lethal foetal akinesis harbouring a homozygous Y259X mutation in the *LMNA* gene were obtained as an autopsy sample after an informed consent and were a kind gift from Prof. Jos Broers. Conditions to induce quiescence and stress-induced premature senescence have been described previously (Pekovic *et al*., 2011). U2OS cells stably expressing GFP-lamin A were selected in G418 (400 μg mL^−1^). MG-132 (Calbiochem, Merck Millipore, Watford, UK) was used at a concentration of 30 μm and added to culture medium for 6 h. Caffeine (Sigma, Gillingham, UK) was added to cells at a concentration of 10 mm for 1 h prior to irradiation. The ATMi KU-55933 (Tocris Bioscience, Abingdon, UK) was used at a final concentration of 10 μm.

### *In situ* extraction, immunocytochemistry, laser microirradiation and microscopy

The sequential treatment of HDF on coverslips with detergents, nucleases and salt was essentially as described (Pekovic *et al*., 2007). Cells were fixed at various times during the extraction procedure and processed for immunofluorescence. Immunofluorescence was carried out according to standard laboratory procedures (Markiewicz *et al*., 2002). Secondary antibodies used were donkey anti-mouse or anti-rabbit IgG conjugated to FITC or TRITC (Stratech, Suffolk, UK) and coverslips were mounted in DAPI, Sigma, Gillingham, UK to visualize DNA. Confocal microscopy was performed using a LSM 510 META, equipped with 40×/1.3 oil-immersion lens and photomultiplier tube. Fluorophore signals were optimized, and images were taken in sequential mode. Images were subsequently adjusted in ImageJ or Photoshop (Adobe, Maidenhead, UK). Laser microirradiation was performed exactly as described (Bekker-Jensen *et al*., 2006).

### Immunochemical methods

For immunoprecipitations, HDF were first cross-linked *in vivo* using DSP (Dithiobis[succinimidyl propionate]) (Pierce, Cramlington, UK), essentially as described (Fujita *et al*., 2002). Briefly, HDF were washed with PBS before adding DSP to a final concentration of 1 mm. Plates were swirled for 15 min at room temperature before the addition of 1 m Tris-HCl pH 7.5 to a final concentration of 25 mm for 15 min at room temperature to quench the reaction. Plates were then washed with PBS, pelleted and lysed in NP-40 buffer (50 mm Tris-HCl pH 7.5, 150 mm NaCl, 1% NP-40, 100 mm EDTA) containing freshly added complete protease and phosphatase inhibitor (Thermo, Fisher Scientific, Cramlington, UK) for 20 min on ice. Lysates then cleared by centrifugation at 10500 × g for 10 min and added to Dynabeads Protein G (Invitrogen, Life Technologies, Paisley, UK) with prebound lamin A/C (sc-7292; Santa Cruz, Heidelberg, Germany) or unspecific IgG overnight at 4 °C. Beads were then washed and boiled for 10 min in sample buffer at 95 °C before loading onto SDS-PAGE gels. For 293T and U2OS immunoprecipitations, cells were lysed in EBC buffer and then extracts were added to GFP-Trap beads (Chromotek, Martinsried, Germany). Beads were washed and bound proteins eluted as for HDF. Antibodies used in this study included mouse monoclonal antibodies to lamin A/C (sc-636; Santa Cruz, Heidelberg, Germany), lamin A/C (Jol2; Immuquest, Middlesbrough, UK), lamin B2 (LN43; Immuquest, Middlesbrough, UK), β-actin (AC-40; Sigma, Gillingham, UK), γ-H2AX (JBW301; Millipore, Watford, UK), HA tag (F-7; Roche, Burgess Hill, UK), HA tag (sc-7392; Santa Cruz, Heidelberg, Germany), GFP (Roche, Burgess Hill, UK), GFP (sc-9996; Santa Cruz, Heidelberg, Germany), ATM (2C1; Genetex, Irvine CA, USA), PCNA (sc-56; Santa Cruz, Heidelberg, Germany); rabbit polyclonal antibodies to 53BP1 (NB100-304; Novus, Cambridge, UK), 53BP1 (sc-22760; Santa Cruz, Heidelberg, Germany), β-tubulin (Abcam, Cambridge, UK ), LAP2α (IQ-175; Immuquest, Middlesbrough, UK), H2A (2578; Cell Signalling, Leiden, The Netherlands), KAP1 (A300-274; Bethyl, Cambridge BioScience; Cambridge, UK), KAP1(S824p) (A300-767; Bethyl, Cambridge BioScience; Cambridge, UK), JMJD2A (NB110-40585; Novus, Cambridge, UK), and goat polyclonal antibody to MCM6 (sc-9843; Santa Cruz, Heidelberg, Germany). For biochemical fractionation experiments, typically 6 × 10 cm plates of 70–80% confluent HDF were collected and subjected to the extraction process as described (Pekovic *et al*., 2007). Pellets were resuspended in hypotonic buffer for 10 min before the addition of sample buffer, whilst sample buffer was added directly to all supernatant fractions. Equal volumes were then loaded for each fraction.

### Colony forming assay

U2OS cells were transfected twice in 24 h with siRNA and then harvested by trypsinization after 48 h from the initial transfection. Cells were plated by serial dilution into 60-mm culture dishes, allowed to adhere overnight and then treated with different doses of IR. Cells were returned to the incubator for 12 days before staining with 6% glutaraldehyde (v/v) and 0.5% crystal violet (wt.vol) in dH_2_O. Dishes were then rinsed and the number of colonies formed counted. The surviving fraction at each IR dose was calculated based on the plating efficiency of the untreated plates.

## Supporting Information

**Fig. S1** (A) HDF were extracted *in situ* as in Fig. B, fixed and immunostained with NuMA and lamin A/C antibodies. (B) Generation of U2OS/GFP-lamin A stable cell line. The level of overexpression of lamin A was assessed by immunoblotting with lamin A/C antibody. WCE, whole cell extract. (C) U2OS/GFP-lamin A cells were fixed and immunostained with 53BP1 antibody. Scale bar, 10 μm.

**Fig. S2** (A) U2OS/GFP-lamin A cells transfected with HA-53BP1 BRCT for 24 h were pretreated with ATMi (10 μm) for 1 h before exposure to IR (10 Gy, 1 h recovery). Cell extracts were then subjected to immunoprecipitation using GFP-Trap beads, and bound complexes were then analysed by immunoblotting using 53BP1 and GFP antibodies. β-tubulin was a loading control. WCE, whole cell extract, IP: immunoprecipitates. WCE represents 1% input. (B) HDF were exposed to IR (2 Gy), fixed 1 h later and immunostained with combinations of the indicated antibodies. Scale bar (10 μm). (C) U2OS cells were treated with scrambled siRNA or siRNA specific for lamins A/C. Whole cell extracts (WCE) were prepared an immunoblotted with antibodies against lamins A/C, 53BP1 or MCM6. The percentage of 53BP1 in siRNA treated WCE is shown.

**Fig. S3** (A) HDF were transfected and CTRL or LMNA siRNA and cell extracts were prepared 72 h later. Protein levels were assessed by immunoblotting with the indicated antibodies. β-tubulin was a loading control. WCE, whole cell extract. Levels of 53BP1 are expressed relative to untreated control siRNA treated HDF. (B) Protein extracts were prepared from proliferating, quiescent or senescent HDF before immunoblotting with the indicated antibodies. (C) Relating to Fig. E. Human dermal fibroblasts and Y259X cells were treated with IR (3 Gy), fixed at the times indicated and fixed and stained with γ-H2AX antibody. (D) WT or Y259X HDF were treated as in Fig. G, then fixed and stained with the indicated antibodies. Scale bar (10 μm). (E) Cell extracts were prepared from WT or Y259X HDF, and levels of KAP1 were analysed by immunoblotting.
