# Supplementary figures and images for: Lamin A/C-dependent interaction with 53BP1 promotes cellular responses to DNA damage
Source: Aging Cell. 2015 Jan 23;14(2):162–9. doi: 10.1111/acel.12258 (PMC4364828; doi:10.1111/acel.12258)

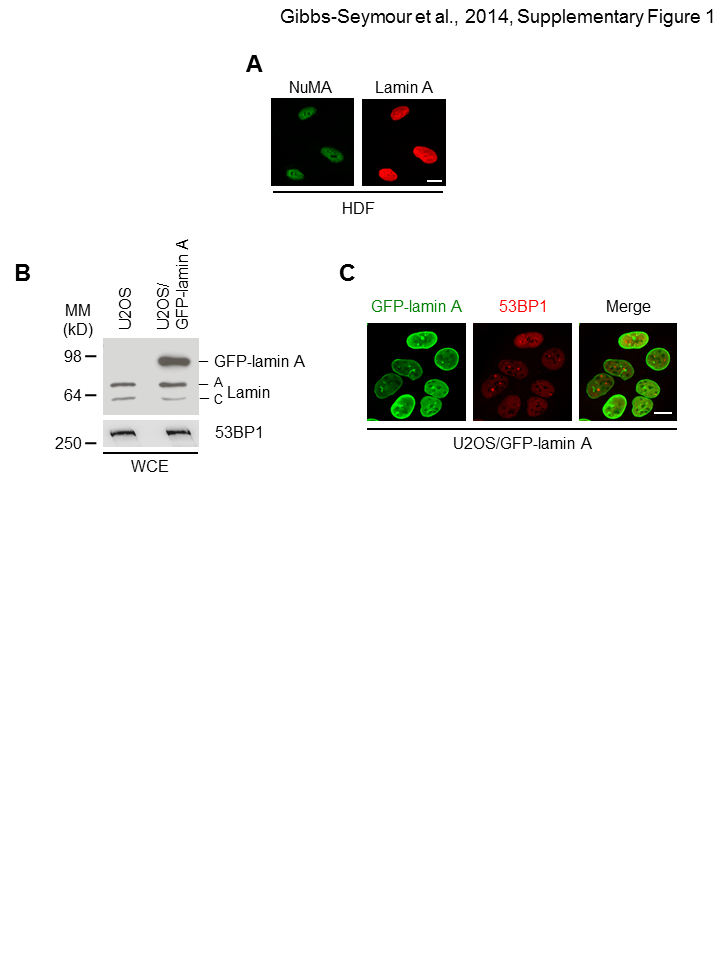

Supplement: Supplementary file 1 [file acel0014-0162-sd1.tif]

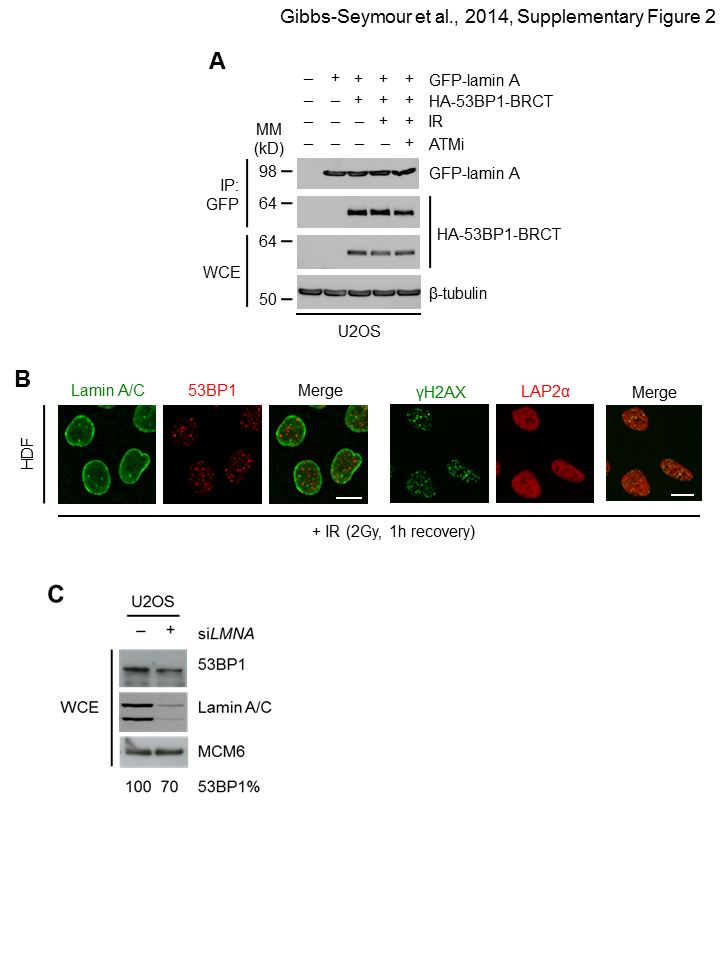

Supplement: Supplementary file 2 [file acel0014-0162-sd2.tif]

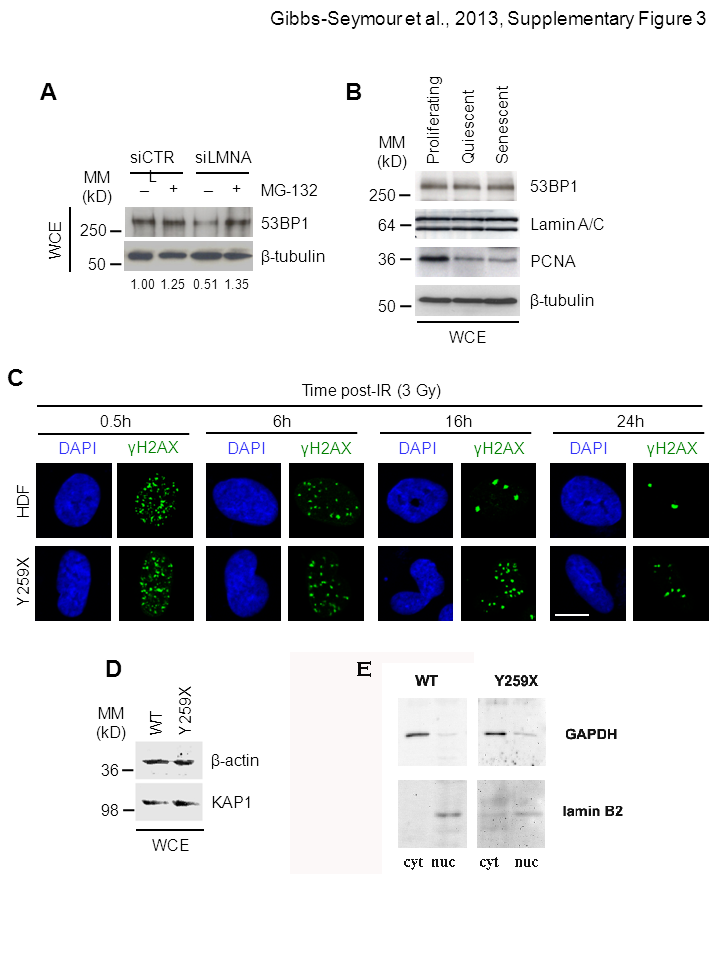

Supplement: Supplementary file 3 [file acel0014-0162-sd3.tif]
